# MEP-Net: a PIDNet-based model with median-enhanced spatial-channel attention for segmentation of hepatocellular carcinoma in CEUS images

**DOI:** 10.1186/s12880-026-02416-x

**Published:** 2026-05-13

**Authors:** Si-Hua Yang, Jing-Bin Wen, Yi-Ran Li, Fang-Fang Zhang, Wei-Qi Li, Shu-Qun Cheng

**Affiliations:** 1 School of Biomedical Engineering, Southern Medical University, Guangzhou, 510515 China; 2 Eastern Hepatobiliary Surgery Hospital, The Second Military Medical University, Shanghai, 200438 China; 3 The First Affiliated Hospital of Wenzhou Medical University, Wenzhou, China; 4 School of Health Science and Engineering, University of Shanghai for Science and Technology, Shanghai, 200433 China

**Keywords:** Hepatocellular carcinoma, Contrast-enhanced ultrasound, Median-enhanced spatial-channel attention, Efficient channel attention, Semantic segmentation

## Abstract

**Purpose:**

Hepatocellular carcinoma (HCC) remains a major global health concern due to its high incidence and mortality. Contrast-enhanced ultrasound (CEUS) offers notable advantages for HCC diagnosis, including real-time imaging and non-invasiveness. However, CEUS images are often affected by noise, uneven signal distribution, and unclear boundaries between lesions and surrounding tissues, which pose challenges for automatic lesion segmentation.

**Methods:**

To improve segmentation performance, this study proposes an improved PIDNet-based model termed MEP-Net. The model integrates a median-enhanced spatial–channel attention mechanism (MECS) and an efficient channel attention (ECA) module to enhance lesion-related feature representation and multi-branch feature fusion. We evaluate the model on a self-built CEUS dataset and the publicly available BUSI breast ultrasound dataset. It is also compared with several mainstream semantic segmentation methods, and ablation studies are conducted to analyze the contribution of each module.

**Results:**

The results show that MEP-Net outperforms the baseline PIDNet by 1.95%, 1.25%, and 2.51% in Dice, MIoU, and Recall, respectively, on the CEUS dataset, and by 1.37%, 1.06%, and 2.41% on the BUSI dataset. In addition, MEP-Net is compared with eight semantic segmentation methods and demonstrates superior overall segmentation performance and improved lesion representation. Ablation studies further confirm the complementary benefits of the MECS and ECA modules in improving segmentation accuracy.

**Conclusion:**

The proposed MEP-Net achieves improved performance in CEUS image segmentation. By introducing attention mechanisms tailored to ultrasound image characteristics, it provides an effective approach for automatic HCC lesion segmentation.

## Introduction

Hepatocellular carcinoma (HCC) ranks among the leading causes of cancer-related deaths worldwide. According to the World Health Organization, its global incidence continues to rise, making it a significant public health concern [1]. Despite ongoing advancements in treatment options—such as surgical resection, liver transplantation, radiofrequency ablation, and targeted therapies—the overall five-year survival rate for HCC remains low, largely because most patients are diagnosed at intermediate or advanced stages [2]. Therefore, enhancing the early detection and accurate localization of HCC is crucial for improving patient outcomes.

Early diagnosis of liver cancer depends greatly on high-quality imaging analysis. At present, commonly used imaging techniques include computed tomography (CT), magnetic resonance imaging (MRI), and ultrasound. Among these, contrast-enhanced ultrasound (CEUS) has been widely used for evaluating liver lesions, owing to its advantages such as real-time imaging, low cost, and lack of ionizing radiation. Compared with conventional ultrasound, CEUS can dynamically reflect the blood perfusion process of liver tissue, providing more information for identifying tumor microvascular characteristics [3]. Previous studies have demonstrated the value of CEUS in assisting HCC diagnosis. For example, Luo et al. [4] developed a logistic regression model that integrates CEUS features with clinical information to achieve non-invasive diagnosis of MTM-HCC. Kondo et al. [5] proposed a machine learning–based method for CEUS image analysis to enable automatic classification of liver lesions.These studies suggest that HCC identification and analysis in CEUS images generally rely on prior localization of lesion regions. In most cases, this step still depends on manual delineation or empirical judgment. Such approaches are time-consuming and subjective, which limits their applicability in large-scale clinical settings. In addition, CEUS images often exhibit speckle noise, low local contrast, and unclear boundaries. HCC lesions also show heterogeneity in enhancement patterns and morphology. These factors further increase the difficulty of accurate lesion segmentation [6].Therefore, developing automated methods for HCC lesion segmentation in CEUS images is important for improving lesion localization efficiency and supporting subsequent quantitative analysis.

In recent years, deep learning methods have achieved significant progress in medical image segmentation and have shown strong potential in various ultrasound segmentation tasks. Previous studies have explored the segmentation of organs or lesions, such as the kidney, thyroid, and breast, in CEUS images. HCC lesion segmentation has also been studied in CT and MRI images. However, research on automatic segmentation of HCC lesions in CEUS images remains limited. Due to substantial differences in imaging mechanisms between CEUS and CT/MRI, existing methods are not directly applicable to this task. In liver CEUS images, HCC lesions often exhibit heterogeneous perfusion patterns, uneven local enhancement, unclear transitions between lesions and surrounding tissues, and interference from speckle noise. These characteristics increase the difficulty of segmentation. Therefore, HCC segmentation in CEUS images requires models that can effectively capture lesion-related semantic features while preserving local structural and detail information. This is particularly important in complex backgrounds where lesion appearance is unstable and contrast is limited. PIDNet [7] adopts a multi-branch parallel architecture to jointly model semantic and detail information. It achieves a good balance between feature representation capability and computational complexity, making it a suitable baseline for this task. However, the original PIDNet is primarily designed for natural scene segmentation. It does not explicitly address challenges in CEUS images, such as speckle noise, local intensity variations, and low contrast between lesions and background. Based on these considerations, this study aims to enhance PIDNet by improving its ability to handle local noise and low-contrast lesion features, while retaining its strength in joint semantic–detail modeling. This is expected to improve its performance in HCC segmentation on CEUS images.

To validate this idea, an improved model, termed MEP-Net, is developed based on PIDNet. While preserving its multi-branch feature modeling capability, two modules are introduced: a median-enhanced channel–spatial module (MECS) and an efficient channel attention (ECA) mechanism. The MECS incorporates median statistical information to better capture the central tendency of channel responses. This design reduces the influence of local abnormal responses during feature recalibration, making the model more robust to speckle noise and unstable local responses commonly observed in CEUS images. The ECA module models inter-channel dependencies to enhance the selection of lesion-related features, thereby improving feature representation for low-contrast and heterogeneous lesion regions. With these enhancements, the model is better suited to handle the characteristics of CEUS images, including low contrast, unclear boundaries, and heterogeneous lesion appearance. Finally, experiments are conducted on a self-collected CEUS dataset and the publicly available BUSI dataset. The performance of the proposed method is evaluated through comparisons with multiple segmentation models and ablation studies.

## Related work

At present, research on automatic segmentation of liver tumors in CEUS images remains limited. Early methods mainly rely on traditional image processing techniques, such as region growing, thresholding, active contour models (ACM), and edge detection [8, 9]. These methods typically extract lesions based on intensity, texture, or edge information, and are sensitive to image quality, parameter settings, and initial conditions. In CEUS images, lesion boundaries are often unclear, local contrast is low, and speckle noise is prevalent. As a result, the performance of these methods is easily affected, leading to suboptimal segmentation results.

With the development of deep learning, medical image analysis has gradually shifted from hand-crafted feature design to data-driven modeling. In the field of CEUS image segmentation, existing studies mainly focus on applications in the breast, thyroid, and kidney. Wan et al. [10]proposed CEUS-Net, which introduces a structure enhancement module into the U-Net framework for breast and thyroid CEUS image segmentation, demonstrating the effectiveness of architectural optimization in CEUS scenarios. Yang et al. [11]further incorporated spatial and temporal information to model renal lesions, improving lesion recognition in CEUS sequences. Chen et al. [12] proposed DPRA-Net, which enhances feature representation of thyroid nodules through a dual-path recursive attention mechanism. In addition, Liu et al. [13]developed Trans-CEUS by combining the Swin Transformer with U-Net, enabling joint segmentation and classification of thyroid CEUS images and highlighting the potential of Transformer-based models in dynamic CEUS analysis. Overall, existing methods improve lesion representation through architectural design, spatiotemporal information fusion, attention mechanisms, and global dependency modeling. Although these approaches have achieved promising results in specific organ scenarios, they mainly focus on general feature enhancement and temporal modeling. As a result, they lack targeted designs for challenges commonly observed in liver CEUS images, such as speckle noise, local abnormal enhancement, and low contrast between lesions and background.

In contrast, research on automatic segmentation of HCC or liver tumors has mainly focused on CT and MRI modalities. Christ et al. [14] proposed a cascaded fully convolutional network (cascaded FCN), which adopts a two-stage strategy by first segmenting the liver and then the tumor, thereby improving overall segmentation accuracy. Li et al. [15] introduced H-DenseUNet, which enhances contextual modeling by combining 2D and 3D features, leading to improved liver tumor segmentation performance. Building on these approaches, attention mechanisms and boundary enhancement strategies have been further explored to improve tumor segmentation. For example, Li et al. [16] proposed Dual Attention U-Net, which enhances the response to tumor regions through a dual attention mechanism. Cao et al. [17] developed BDense-UNet, which improves tumor boundary modeling through a boundary-aware design. Lin et al. [18] proposed AIM-Unet, which combines attention mechanisms with Inception modules to enhance feature extraction for complex lesion regions. These methods provide valuable insights into contextual modeling, salient region selection, and boundary enhancement. However, they are primarily developed for CT and MRI data. Due to substantial differences between CEUS and CT/MRI in imaging mechanisms, noise characteristics, and lesion enhancement patterns, the applicability of these methods to HCC segmentation in CEUS images remains to be further validated.

Overall, existing studies improve segmentation performance through salient region enhancement, global dependency modeling, and detailed structural representation. However, for the specific task of HCC segmentation in CEUS images, there is still a lack of joint modeling for challenges such as speckle noise, unstable local responses, and low contrast between lesions and background. To address these issues, this study introduces the MECS and ECA mechanisms into the PIDNet framework to improve the model’s ability to represent complex response distributions and low-contrast lesion features in CEUS images.

## Dataset and preprocessing

### Dataset

This study uses two datasets for model training and evaluation: a CEUS image dataset provided by the Eastern Hepatobiliary Surgery Hospital of Naval Medical University, and the publicly available BUSI breast ultrasound dataset. The former is used for training, validation, and primary testing, while the latter serves as an external test set to evaluate the adaptability and robustness of the proposed model across different types of ultrasound images.

#### Private CEUS dataset

This dataset was collected from the Department of Ultrasound at the Eastern Hepatobiliary Surgery Hospital. It includes CEUS video data from 283 patients with pathologically confirmed HCC, acquired between December 2021 and August 2024. All data were approved by the institutional ethics committee. The inclusion criteria were as follows: (1) confirmed diagnosis of HCC; and (2) availability of complete CEUS video sequences. The exclusion criteria were: (1) low image quality or severe motion artifacts that hinder clear delineation of lesion boundaries; and (2) presence of other space-occupying liver lesions that may affect liver structure, such as hemangiomas or extensive cirrhotic nodules. A preliminary statistical analysis of the sample size indicates that the dataset provides sufficient statistical power for evaluating segmentation performance on the training and test sets.

This study adopts a patient-level data splitting strategy. The 283 cases are first divided into training, validation, and test sets at the patient level (226, 28, and 29 cases, respectively). Frame extraction and image preprocessing are then performed within each subset. All frames from the same patient are assigned to a sin gle subset, with no overlap across subsets, thereby avoiding data leakage caused by including frames from the same patient in both training and test sets.

After completing patient-level partitioning, each original video segment was downsampled at 1 frame per second (fps) to reduce temporal redundancy. Considering that the contrast agent exhibits dynamic perfusion and washout processes over time during CEUS imaging, resulting in substantial variations in image information density across different time points, we further selected key frames based on the standard deviation of inter-frame grayscale changes. Specifically, the top k frames (k = 10) with the largest grayscale variations in each video segment were identified as key frames, thereby preserving the time points with relatively concentrated information changes during the contrast process and reducing the interference of redundant frames on subsequent learning. On this basis, frames with severe blurring or noise interference were further removed from the selected key frames to improve overall data quality.

The final dataset consists of 2,135 high-quality CEUS images, including 1,728, 193, and 214 images in the training, validation, and test sets, respectively. All images are annotated at the pixel level by radiologists with more than five years of experience. The annotations are further reviewed by at least two ultrasound experts to ensure accuracy and consistency.

#### Public BUSI dataset

The BUSI dataset [19], provided by Assiut University, is a publicly available resource commonly used for breast tumor segmentation in ultrasound images. It comprises three types of images: benign, malignant, and normal. Since the normal images do not contain any annotations—their masks are completely black—only the benign and malignant images were used in this study. A total of 630 images with valid pixel-level lesion masks were selected. Due to differences in imaging characteristics between BUSI and liver CEUS, this dataset is used as a supplementary test set to further evaluate the model’s performance on heterogeneous ultrasound segmentation tasks. Details on dataset access can be found in the Breast Ultrasound Images Dataset (Breast Ultrasound Images Dataset).

### Data preprocessing

**Fig. 1:**
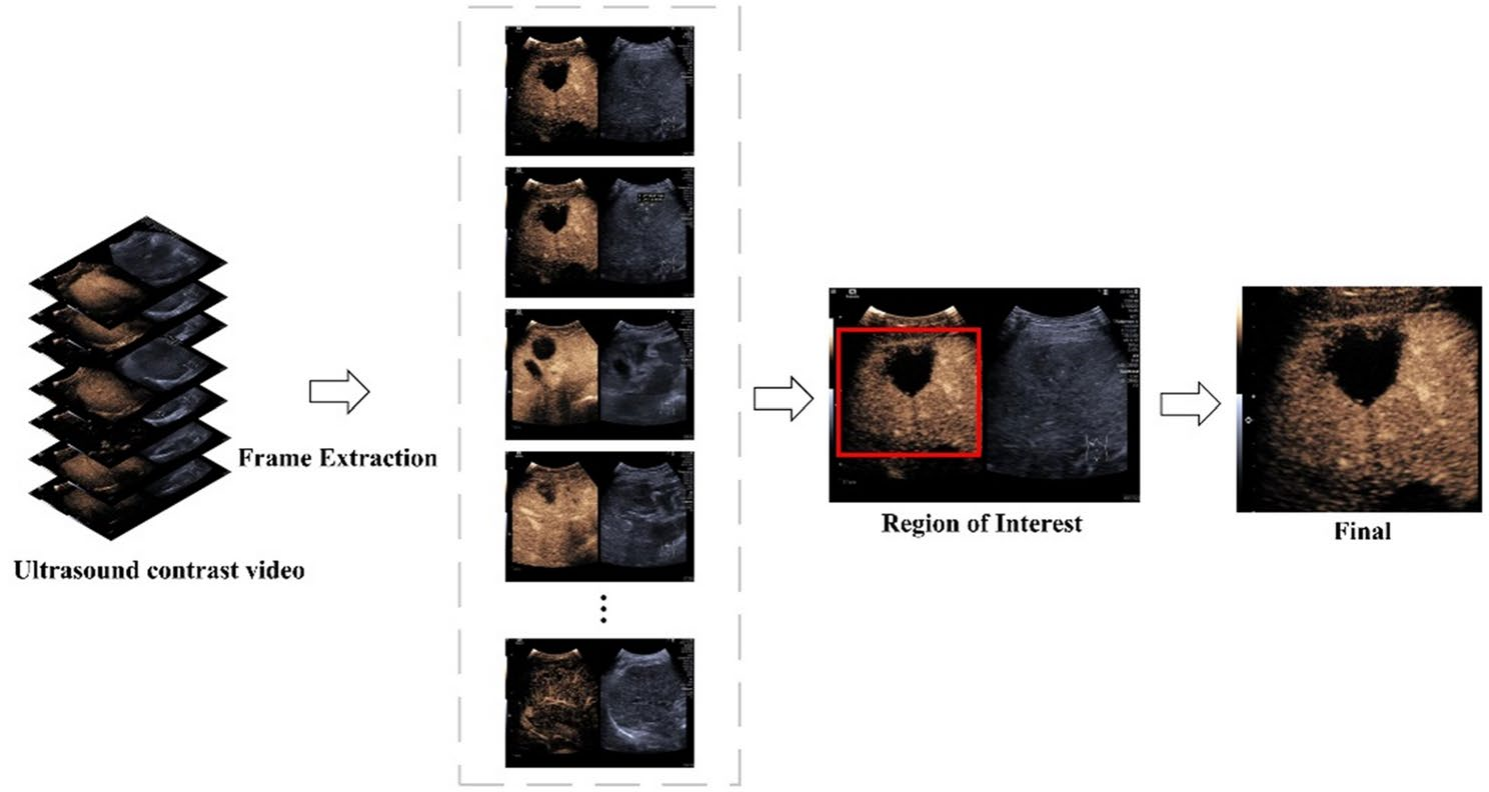
Image preprocessing workflow: cropping the region of interest, excluding background regions, and resizing to a standardized input size

The preprocessing workflow is illustrated in Fig. 1. To ensure a consistent input format, identical preprocessing steps were applied to both datasets. First, the region of interest (ROI) was cropped from each image, and all images were resized to 512 × 512 pixels. Subsequently, intensity normalization was performed. Since the BUSI images are grayscale, each was duplicated into three channels to meet the input size requirements of the model. To improve the model’s generalization and reduce overfitting, several data augmentation techniques were applied to the training set, including random rotation (± 15°), horizontal flipping, brightness adjustment, and gamma correction.

## Method

### Model framework

**Fig. 2:**
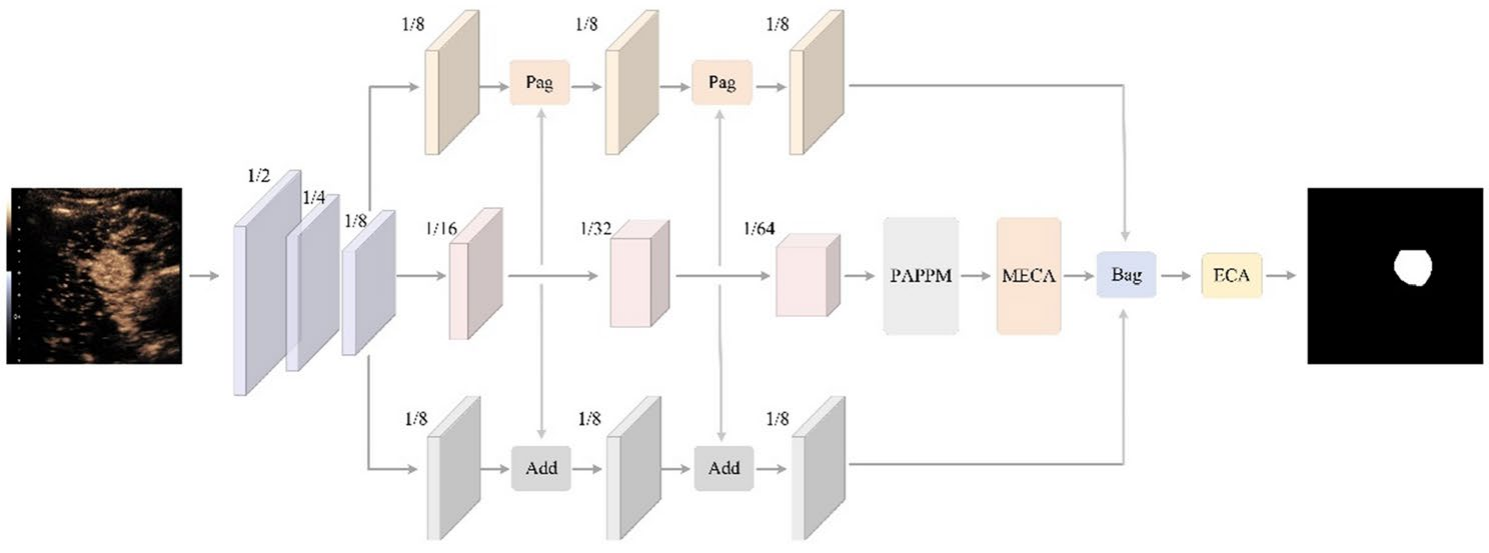
Architecture of the proposed MEP-Net model

This study builds upon the classical three-branch semantic segmentation network PIDNet [7] and proposes an improved architecture, termed MEP-Net, for lesion segmentation in CEUS images of HCC MEP-Net retains the three-branch design of PIDNet to separately model spatial details, semantic context, and boundary-related information. Given that HCC CEUS images often suffer from issues such as speckle noise, local strong echo or artifact interference, uneven distribution of enhanced signals, and unclear lesion boundaries, the network is prone to being affected by abnormal responses, unstable channel responses, and spatial localization deviations during feature extraction and multi-branch fusion. To address this, this paper proposes improvements based on the original three-branch structure, aiming to enhance the representational stability of CEUS lesion regions by strengthening feature statistical description, spatial attention modeling, and fused feature recalibration. The overall architecture of the proposed model is illustrated in Fig. 2.

The input image is initially processed by a convolutional layer to extract low-level features, which are then fed into three parallel branches for multi-dimensional feature encoding. In the original architecture, the P branch incorporates a pixel attention guidance module (Pag) [7], which leverages semantic information from the I branch to help it focus on more discriminative regions and better capture lesion details. The I branch employs a multi-scale feature aggregation strategy to integrate local and global semantic context across layers. It also integrates a lightweight parallel pyramid pooling module (PAPPM) [7] in deeper layers to enhance the modeling of long-range dependencies. The D branch is used to capture high-frequency structural features and provides additional detail cues for the final feature fusion.

Based on this, two targeted improvements are made to PIDNet in this paper. First, after the PAPPM module in the I branch, the MECS module is added to jointly recalibrate the channel and spatial responses at the high-level semantic feature layer. This design primarily addresses issues such as local intensity fluctuations and abnormal responses in CEUS images, enhancing the expression of lesion-related semantic features. Second, after the features from the three branches are fused through the boundary attention-guided module (Bag), the ECA module is introduced to perform lightweight channel recalibration on the fused features, further coordinating the response relationships between spatial details, semantic context, and boundary information. Finally, the fused features are upsampled to restore the original image resolution and output a pixel-level probability map of the lesion region.

### MECS module

**Fig. 3:**
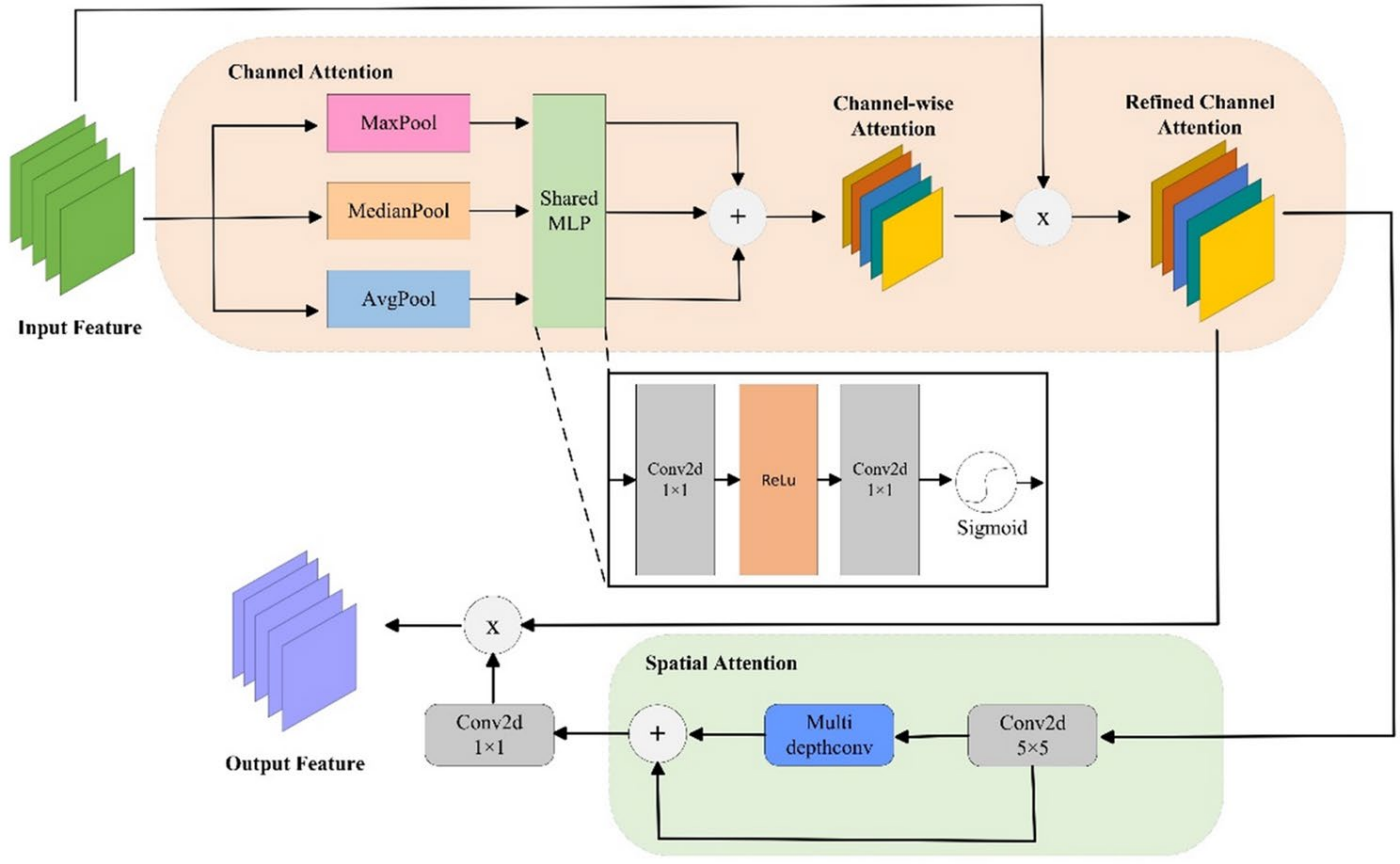
Architecture of the proposed MECS module, which integrates channel attention and spatial attention mechanisms to enhance feature representation

For HCC CEUS images, the microbubble-enhanced signals in the lesion region often show non-uniform distribution and are affected by factors such as speckle noise, local strong echoes, and blurred boundaries. These issues can lead to local abnormal responses or spatial localization errors in the high-level semantic features extracted by the network. Simple statistical measures or single-scale spatial modeling are insufficient to fully capture such complex feature distributions. Therefore, this paper proposes the MECS module, which combines mean, maximum, and median statistical information to model the channel response distribution. It also enhances the spatial representation of lesions with different sizes and boundary shapes by using multi-scale spatial attention. The module consists of a channel attention submodule and a spatial attention submodule, which improve the discriminative expression related to the lesion in both the channel and spatial dimensions. Its structure is shown in Fig. 3.

#### Channel attention submodule

In CEUS images, characteristics such as speckle noise, local strong echo interference, and unstable spatial signal distribution may affect the assessment of channel-wise feature importance. Existing channel attention methods, such as SENet [20] and BAM [21], typically generate channel descriptors based on global average pooling (GAP) or global max pooling (GMP). GAP reflects the overall response distribution but may smooth local salient differences, whereas GMP focuses on strong responses but is sensitive to local extremes. In medical imaging scenarios, where structural details, noise components, and abnormally high responses may coexist, relying on a single statistical measure is often insufficient to fully characterize channel feature distributions [22]. CBAM [23] improves channel modeling by combining GAP and GMP, but it does not utilize the median statistic, which reflects the central tendency of the distribution. Based on this, this paper introduces Global Median Pooling (GMedP) into the channel attention design and combines it with GAP and GMP to construct the channel descriptor. Specifically, GAP reflects the overall average response, GMP emphasizes local significant responses, while GMedP provides a relatively robust description of the central tendency. The combination of the three helps characterize the channel response distribution more comprehensively, reducing the reliance of channel weight estimation on a single extreme response, thus providing supplementary statistical information for feature recalibration in CEUS images with prominent local strong responses and grayscale fluctuations.

Specifically, let the input feature map be $\:F\in\:{\mathrm{R}}^{C\times\:H\times\:W}$. First, global average pooling, global max pooling, and global median pooling are applied to $\:F$, generating three channel descriptors $\:{F}_{\mathrm{avg}}$, $\:{F}_{\mathrm{max}}$, and $\:{F}_{\mathrm{med}}$, where

1 $$\:\begin{array}{c}{F}_{avg},{F}_{max},{F}_{med}={R}^{C\times\:H\times\:W}\end{array}$$

These descriptors are then fed into a shared multilayer perceptron (MLP) for nonlinear mapping. The MLP consists of two $\:1\times\:1$ convolutional layers with a ReLU activation in between. The first convolution reduces the channel dimension from $\:C$ to $\:C/r$, and the second restores it to $\:C$. The outputs of the three branches are activated by the Sigmoid function and summed element-wise to obtain the channel response map $\:{F}_{c}$:

2 $$\:\begin{array}{c}{F}_{c}=\sigma\:\left(MLP\left({F}_{avg}\right)\right)+\sigma\:\left(MLP\left({F}_{max}\right)\right)+\sigma\:\left(MLP\left({F}_{med}\right)\right)\end{array}$$

Finally, $\:{F}_{c}$ is multiplied element-wise with the input feature map $\:F$ to produce the refined feature representation $\:{F}^{{\prime\:}}$:

3 $$\:\begin{array}{c}{F}^{{\prime\:}}={F}_{c}\odot\:F\end{array}$$

Here, *σ* denotes the Sigmoid function, and ⊙ represents element-wise multiplication.

#### Spatial attention submodule

In HCC CEUS images, there are significant individual differences in lesion size, shape, enhancement range, and boundary transition features, resulting in variable spatial scales and response ranges of the lesion-related regions in the feature map. Spatial modeling with a single receptive field is insufficient to fully represent the complex spatial distribution characteristics. Previous studies have shown that multi-scale receptive field structures can enhance the ability of medical image segmentation models to represent lesions with diverse morphologies [24, 25]. Therefore, a multi-scale depthwise separable convolution structure is introduced in the spatial attention submodule to enhance the model’s ability to model spatial information for lesions of different sizes and boundary transition areas.

Specifically, the channel-refined feature map $\:{F}^{{\prime\:}}$ is first processed by a $\:5\times\:5$ depthwise convolution to extract basic spatial features, denoted as $\:{F}_{b}$. Then, $\:{F}_{b}$is fed into multiple depthwise convolution branches with different receptive fields to capture multi-scale spatial responses. The outputs of these branches are summed element-wise to obtain the fused spatial feature map $\:{F}_{s}$. A $\:1\times\:1$ convolution is then applied to generate the spatial attention map, which is multiplied element-wise with $\:{F}^{{\prime\:}}$ to produce the final output feature map $\:{F}^{{\prime\:}{\prime\:}}$:

4 $$\:\begin{array}{c}{F}_{b}={D}_{5\times\:5}\left({F}^{{\prime\:}}\right)\end{array}$$

5 $$\:\begin{array}{c}{F}_{s}=\sum\:_{i=1}^{n}{D}_{i}\left({F}_{b}\right)\end{array}$$

6 $$\:\begin{array}{c}{F}^{{\prime\:}{\prime\:}}=C{\mathrm{o}\mathrm{n}\mathrm{v}}_{1\times\:1}\left({F}_{s}\right)\odot\:{F}^{{\prime\:}}\end{array}$$

where $\:{D}_{5\times\:5}(\cdot\:)$ denotes the $\:5\times\:5$ depthwise convolution, $\:{D}_{i}(\cdot\:)$ represents depthwise convolution with different receptive fields, $\:n$ is the number of convolution branches, $\:{\mathrm{Conv}}_{1\times\:1}$ denotes a $\:1\times\:1$ convolution, and $\:\odot\:$ indicates element-wise multiplication.

### ECA module

In MEP-Net, the Bag module is used to fuse spatial details, semantic context, and boundary-related features from the P, I, and D branches. For HCC CEUS images, factors such as blurred lesion boundaries, uneven local enhancement, and background tissue interference may cause redundancy or inconsistency in the feature responses output by different branches. Directly using the fused features for final prediction may affect the effective expression of lesion-related information. Based on this, the ECA module [26] is introduced after the Bag module to model local channel dependencies through lightweight channel recalibration, further enhancing the coordination and discriminative expression of the fused features.

**Fig. 4:**
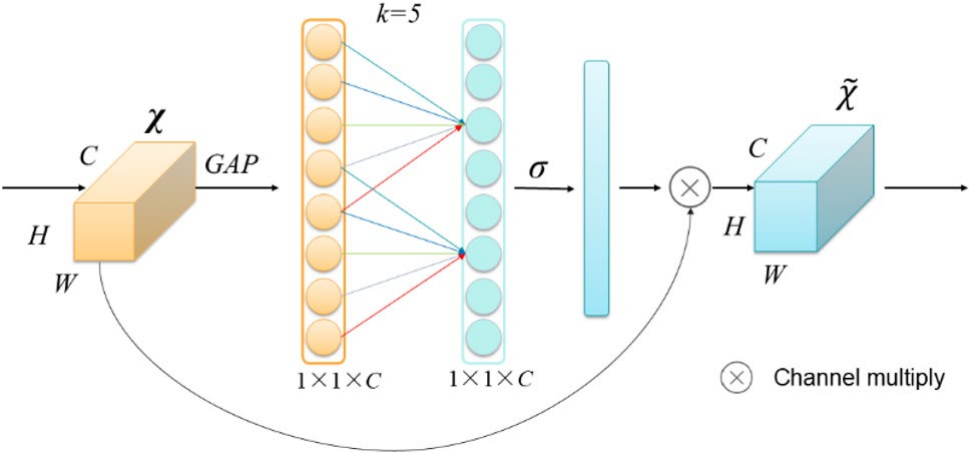
Structure of the Efficient Channel Attention (ECA) module

The core idea of the ECA module is to avoid explicit dimensionality reduction and instead model local inter-channel dependencies directly on the original channel dimension. As illustrated in Fig. 4, the ECA module operates as follows: first, global average pooling is applied to the input feature map $\:F\:\in\:\:{R}^{C\times\:H\times\:W}$ to produce a channel descriptor vector $\:z\:\in\:\:{R}^{C}$; then, a one-dimensional convolution with kernel size $\:k$ is used to capture local cross-channel interactions, generating an attention weight vector $\:a\:\in\:{\:R}^{C}$; finally, the attention weights are activated by a sigmoid function and used to reweight the original feature map along the channel dimension, yielding the enhanced output feature map $\:{F}^{{\prime\:}}$. The corresponding computation is as follows:

7 $${z_c} = {1 \over {H \times W}}\mathop \sum \limits_{i = 1}^H \mathop \sum \limits_{j = 1}^W {F_c}\left( {i,j} \right)\ ,\forall c \in \left\{ {1,2, \ldots ,C} \right\}$$

8 $$\:\begin{array}{c}a=\sigma\:\left(\mathrm{C}\mathrm{o}\mathrm{n}\mathrm{v}1{\mathrm{D}}_{\mathrm{k}}\left(z\right)\right)\end{array}$$

9 $$\:\begin{array}{c}{F}_{c}^{{\prime\:}}={a}_{c}\cdot\:{F}_{c}\ ,\forall\:c\in\:\{\mathrm{1,2},\dots\:,C\}\end{array}$$

Here, σ denotes the Sigmoid activation function, and *k* represents the kernel size of the one-dimensional convolution, which is typically determined adaptively based on the number of channels *C*. Through this lightweight local channel interaction modeling, the ECA module adaptively adjusts the responses of important channels in the fused features, thereby enhancing the coordination and discriminative representation of multi-branch features.

## Experiments and results

### Experimental setup and parameters

The proposed MEP-Net model employs ResNet-18 [27] as its encoder backbone initialized with pretrained weights from the ImageNet dataset to improve convergence speed and generalization performance. Considering the model size, dataset scale, and hardware conditions, the number of training epochs is set to 200, and the batch size is set to 8. The initial learning rate is set to 0.001 to balance training stability and optimization efficiency. Stochastic gradient descent (SGD) is used as the optimizer, with a momentum of 0.9 and a weight decay of 0.0005. The momentum term helps reduce oscillations during optimization and accelerates convergence, while weight decay mitigates overfitting and improves generalization. To further enhance convergence stability in the later stages of training, a multi-stage learning rate decay strategy is adopted, which dynamically adjusts the learning rate at each iteration. This allows faster learning in the early stages and more stable parameter optimization in the later stages.

All experiments were implemented using the PyTorch framework and conducted on a workstation equipped with an NVIDIA RTX 3090 GPU to ensure both training efficiency and reproducibility.

### Loss function

In this study, the same loss function as that used in the baseline PIDNet was adopted. This loss consists of an auxiliary semantic loss, a boundary supervision loss, a semantic segmentation loss, and a boundary-aware semantic loss. These components are jointly used to optimize both the semantic segmentation and boundary detection tasks.Specifically, an auxiliary semantic head was added to the output of the first Pag module to generate an additional semantic loss $\:{l}_{0}$. This loss helps optimize the entire network. For the boundary branch, a weighted binary cross-entropy loss $\:{l}_{1}$ was used to supervise boundary detection. This design helps alleviate the class imbalance between boundary and non-boundary pixels. Meanwhile, $\:{l}_{2}$ denotes the cross-entropy loss used for the main segmentation branch. To further coordinate the semantic segmentation and boundary detection tasks, $\:{l}_{3}$ was defined as a boundary-aware cross-entropy loss. This loss uses the output of the boundary head to constrain the segmentation prediction. It is formulated as follows:

10 $$\:\begin{array}{c}{l}_{3}=-\sum\:_{i,c}1\left({b}_{i}>t\right){s}_{ic}^{gt}\mathrm{log}{s}_{ic}\end{array}$$

where $\:t$ is a predefined threshold, $\:{b}_{i}$ denotes the output of the boundary head for the $\:i$-th pixel, and $\:{s}_{ic}^{gt}$ and $\:{s}_{ic}$ represent the ground-truth label and predicted probability for class $\:c$, respectively. $\:1(\cdot\:)$ denotes the indicator function.

Accordingly, the overall loss function is defined as:

11 $$\:\begin{array}{c}L={\lambda\:}_{0}{l}_{0}+{\lambda\:}_{1}{l}_{1}+{\lambda\:}_{2}{l}_{2}+{\lambda\:}_{3}{l}_{3}\end{array}$$

where $\:{\lambda\:}_{0}=0.4$, $\:{\lambda\:}_{1}=20$, $\:{\lambda\:}_{2}=1$, and $\:{\lambda\:}_{3}=1$. The threshold $\:t\:$ was set to 0.8.

### Evaluation metrics

To comprehensively evaluate the performance of the proposed model in segmenting HCC in CEUS images, four widely used evaluation metrics are employed: Dice coefficient, mean Intersection over Union (mIoU), Precision, and Recall. These metrics, commonly used in medical image segmentation tasks, assess the similarity and consistency between the predicted segmentation and the ground truth from multiple perspectives. All metrics range from 0 to 1, with higher values indicating better segmentation performance.

- **Dice coefficient**: Measures the overlap between the predicted region and the actual lesion. It is useful for segmenting small or irregularly shaped targets in medical images.
- **MIoU** : Computes the ratio of the intersection to the union of the predicted and ground truth regions. It is a standard metric in image segmentation.
- **Precision**: Indicates the proportion of correctly predicted lesion pixels among all predicted lesion pixels, reflecting prediction accuracy.
- **Recall**: Represents the proportion of actual lesion pixels that are correctly detected, indicating the model’s ability to find all lesions.

The mathematical definitions of these metrics are shown below:

12 $$\:\begin{array}{c}Dice=\frac{2TP}{FP+2TP+FN}\end{array}$$

13 $$\:\begin{array}{c}MIoU=\frac{TP}{TP+FP+FN}\end{array}$$

14 $$\:\begin{array}{c}Precision=\frac{TP}{TP+FP}\end{array}$$

15 $$\:\begin{array}{c}Recall=\frac{TP}{TP+FN}\end{array}$$

Here, True Positive (TP) refers to lesion pixels correctly predicted by the model; False Positive (FP) denotes non-lesion pixels incorrectly predicted as lesions; and False Negative (FN) indicates lesion pixels that are incorrectly predicted as non-lesions.

### Results

#### Evaluation on private CEUS dataset

To assess the performance of the proposed MEP-Net in the segmentation task, a series of comparative experiments were conducted on a self-constructed CEUS dataset. The model was evaluated against several well-established methods, including FCN [28], U-Net [29], PSPNet [30], DeepLabv3 [31], DeepLabv3+ [32], HRNet [33], LR-ASPP [34], SegFormer [35], and the original PIDNet.

**Table 1:** Performance comparison of segmentation models on the CEUS dataset (mean ± SD)

Model	Precision (%)	Recall (%)	MIoU (%)	Dice (%)
FCN	**83.19 ± 0.50**	50.53 ± 0.65	72.19 ± 0.31	62.87 ± 0.64
U-Net	77.48 ± 1.01	63.86 ± 0.73	76.08 ± 0.28	70.01 ± 0.34
PSPNet	79.16 ± 0.32	61.31 ± 0.27	75.40 ± 0.50	69.10 ± 0.26
DeepLabV3	81.29 ± 0.53	57.96 ± 0.76	74.98 ± 0.21	67.67 ± 0.38
DeepLabV3+	81.09 ± 1.26	56.98 ± 1.14	74.38 ± 0.21	66.91 ± 0.38
LR-ASPP	71.38 ± 1.28	55.34 ± 0.53	71.77 ± 0.10	62.33 ± 0.16
HRNet	82.44 ± 0.84	60.14 ± 0.82	75.71 ± 0.22	69.54 ± 0.25
SegFormer	80.24 ± 0.79	65.03 ± 0.24	77.36 ± 0.17	71.84 ± 0.26
PIDNet	81.42 ± 0.96	65.16 ± 1.28	77.71 ± 0.31	72.38 ± 0.49
MEP-Net	82.43 ± 0.74	**67.67 ± 0.39**	**78.96 ± 0.25**	**74.33 ± 0.38**

Table 1 presents a quantitative comparison of different semantic segmentation models on the CEUS dataset. The evaluation metrics include Precision, Recall, MIoU, and Dice coefficient, with all experiments repeated three times, and the results are expressed as the mean ± standard deviation. Overall, models show a trade-off between Precision and Recall. Traditional CNN methods (e.g., FCN, DeepLab series) generally achieve high Precision but have significantly lower Recall, leading to insufficient coverage of target regions. For example, FCN achieves a Precision of 83.19%, but its Recall is only 50.53%, which limits the overall MIoU and Dice performance and indicates its limited ability to represent complex CEUS dynamic perfusion scenarios.

In contrast, methods such as U-Net, PSPNet, and HRNet show improved Recall, but their Dice and MIoU still have room for improvement, suggesting limitations in region consistency and boundary localization. Among these, U-Net is relatively balanced across all metrics, but its Precision is only 77.48%, indicating some shortcomings in reducing false positives. SegFormer and PIDNet, on the other hand, enhance segmentation performance by improving global modeling or introducing multi-branch structures, achieving better Recall. SegFormer reaches a Recall of 65.03%, close to MEP-Net, but its MIoU and Dice still lag behind, showing that MEP-Net performs better in region consistency. PIDNet improves Dice to 72.38%, reflecting improvements in local details, but it has not fully balanced Precision and Recall.

Based on this, MEP-Net shows more balanced performance across all metrics, maintaining a high Precision (82.43%) while increasing Recall to 67.67%, and achieving the best results in MIoU (78.96%) and Dice (74.33%). Compared to FCN, MEP-Net improves Recall by more than 17% points, with only a slight drop in Precision, indicating that it expands target coverage while controlling false positives. Compared to the baseline PIDNet model, MEP-Net improves Recall, MIoU, and Dice by 2.51%, 1.25%, and 1.95%, respectively, reflecting improvements in regional completeness. These performance gains are likely due to the feature reweighting mechanism, which models spatial and channel information collaboratively, enhancing responses in key regions and improving region consistency to some extent.

Overall, MEP-Net shows balanced and stable performance across all metrics in the CEUS segmentation task. While the improvements are relatively modest, the introduced attention modules demonstrate positive effects in modeling lesion regions.

**Table 2:** Statistical analysis results of segmentation performance between MEP-Net and PIDNet

Metric	PIDNet	MEP-Net	Difference	95% Confidence Interval	*p*-value
Precision / %	81.42 ± 0.96	82.43 ± 0.74	1.03	[-0.27, 2.44]	0.118
Recall / %	65.16 ± 1.28	67.67 ± 0.39	2.51	[0.04, 5.42]	0.046
mIoU / %	77.71 ± 0.31	78.96 ± 0.25	1.25	[0.20, 2.50]	0.0196
Dice / %	72.38 ± 0.49	74.33 ± 0.37	1.95	[0.31, 3.99]	0.0192

Note: The results are presented as the mean ± standard deviation from 3 independent runs. The difference represents the performance improvement of MEP-Net over PIDNet. Statistical analysis was performed using paired bootstrap resampling (5000 resamples), with 95% confidence intervals and p-values estimated accordingly

As shown in Table 2, MEP-Net outperforms PIDNet in metrics such as Precision, Recall, mIoU, and Dice. Specifically, the improvement in Precision is 1.03%, but its confidence interval includes 0 ([-0.27, 2.44]) and the p-value is 0.118, indicating no significant difference. In contrast, the improvements in Recall, MIoU, and Dice are 2.51%, 1.25%, and 1.95%, respectively, with confidence intervals that do not include 0 and p-values all less than 0.05, showing statistically significant differences. In conclusion, except for Precision, MEP-Net demonstrates a significant performance advantage in other key metrics, validating its stable and effective improvements.

**Fig. 5:**
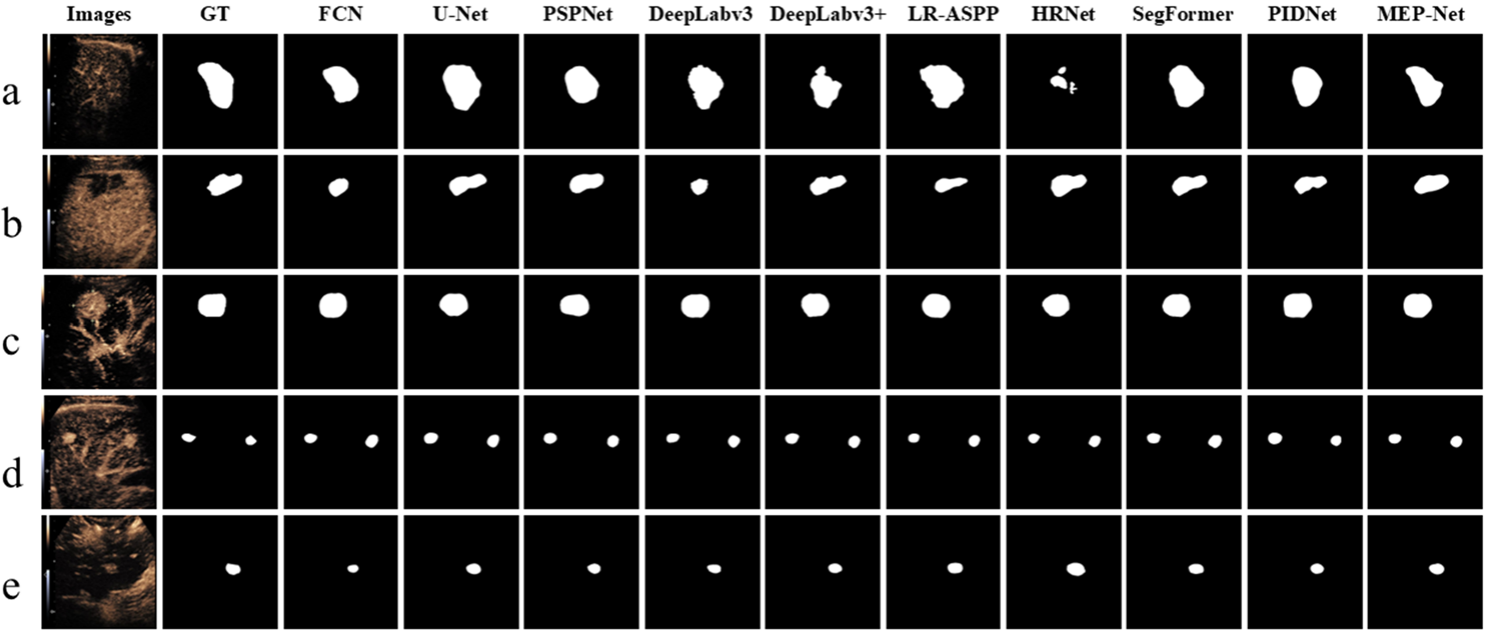
Visual comparison of segmentation results from different models on representative CEUS test images. Each row shows a CEUS image, the corresponding ground truth (GT) mask, and predictions from the compared models

As shown in Fig. 5, five contrast-enhanced ultrasound (CEUS) images are selected from the test set. The selected cases represent a variety of lesion types, including well-defined, blurred, small, and multifocal lesions. In Fig. 5a, the lesion is relatively large and blurry. Most models, including FCN, PSPNet, the DeepLabv3 series, PIDNet, and SegFormer, exhibited substantial under-segmentation. HRNet performed the worst, while U-Net and LR-ASPP tended to over-segment the lesion. In contrast, MEP-Net provided a more accurate reconstruction of the lesion shape, producing results closer to the ground truth. In Fig. 5b, where the lesion is darker and moderate in size, FCN, DeepLabv3, and LR-ASPP failed to fully capture the lesion. Other models produced relatively better results, though slight deviations remained. In Fig. 5c, the lesion is bright and moderate in size, and all models performed similarly with minimal differences. Figure 5d shows multiple bright lesions, and all models produced comparable results in terms of shape and size. In Fig. 5e, the lesion is small and has low contrast. Apart from FCN, most models performed similarly, with MEP-Net showing better detail preservation. Overall, MEP-Net delivered consistent segmentation performance across different lesion types and demonstrated better adaptability and detail retention compared to other models.

#### Evaluation on public BUSI dataset

To further evaluate the performance of MEP-Net on different ultrasound datasets, additional experiments are conducted on the BUSI breast ultrasound dataset. This dataset contains lesions with diverse shapes and varying degrees of boundary ambiguity, making the segmentation task challenging. Since BUSI is a breast ultrasound dataset and differs from liver CEUS in imaging characteristics and lesion presentation, this experiment is mainly used to provide a supplementary evaluation of the model’s performance on heterogeneous ultrasound images.

**Table 3:** Performance comparison of segmentation models on the BUSI dataset (mean ± SD)

Model	Precision (%)	Recall (%)	MIoU (%)	Dice (%)
FCN	**89.80 ± 1.05**	68.63 ± 0.08	80.01 ± 0.30	77.80 ± 0.38
U-Net	84.16 ± 0.10	74.52 ± 0.92	80.82 ± 0.35	79.04 ± 0.48
PSPNet	86.58 ± 0.83	75.13 ± 0.25	81.93 ± 0.20	80.45 ± 0.24
DeepLabV3	87.65 ± 3.32	72.15 ± 0.52	80.96 ± 0.84	79.13 ± 1.04
DeepLabV3+	86.81 ± 1.03	73.85 ± 0.50	81.44 ± 0.17	79.80 ± 0.20
LR-ASPP	82.30 ± 3.34	70.21 ± 1.78	78.37 ± 0.64	75.73 ± 0.83
HRNet	86.50 ± 1.52	76.98 ± 2.09	82.70 ± 0.37	81.44 ± 0.50
SegFormer	89.31 ± 0.19	73.74 ± 0.31	82.23 ± 0.21	80.78 ± 0.26
PIDNet	85.87 ± 1.70	76.15 ± 1.60	82.13 ± 1.21	80.71 ± 1.55
MEP-Net	85.95 ± 1.45	**78.56 ± 0.57**	**83.19 ± 0.41**	**82.08 ± 0.49**

Table 3 presents a comparison of segmentation performance across different methods on the BUSI dataset. A trade-off between Precision and Recall can be observed for each model. FCN and SegFormer achieve high Precision (89.80% and 89.31%, respectively), but their Recall is relatively low (68.63% and 73.74%). This indicates that these models tend to adopt a more conservative prediction strategy, potentially affecting the complete coverage of lesion areas. In contrast, models like U-Net, HRNet, and PIDNet improve Recall, but their Precision decreases accordingly, suggesting that while they expand region coverage, they may introduce additional false positives.

On this basis, MEP-Net shows distinct performance characteristics. It maintains a moderate Precision (85.95%) while achieving the highest Recall (78.56%) among all the methods. It also performs optimally in the Dice (82.08%) and MIoU (83.19%) metrics. Compared to FCN, which has the highest Precision, MEP-Net’s Precision decreases by about 3.85%, but its Recall improves by nearly 10% points, demonstrating a more balanced performance between target region coverage and false positive control. Compared to PIDNet, MEP-Net shows improvements of 2.41%, 1.06%, and 1.37% in Recall, MIoU, and Dice, respectively, further highlighting its improvements in target region coverage. It is also observed that although SegFormer and HRNet perform similarly to MEP-Net in some metrics, they still show a gap in Dice and MIoU. Additionally, HRNet exhibits more performance fluctuations, suggesting room for improvement in region consistency.

Although MEP-Net shows promising results, its Precision is still approximately 3–4% points lower than that of FCN and SegFormer. This suggests that false positives may still occur in complex backgrounds, indicating areas for potential improvement in future work.

**Fig. 6:**
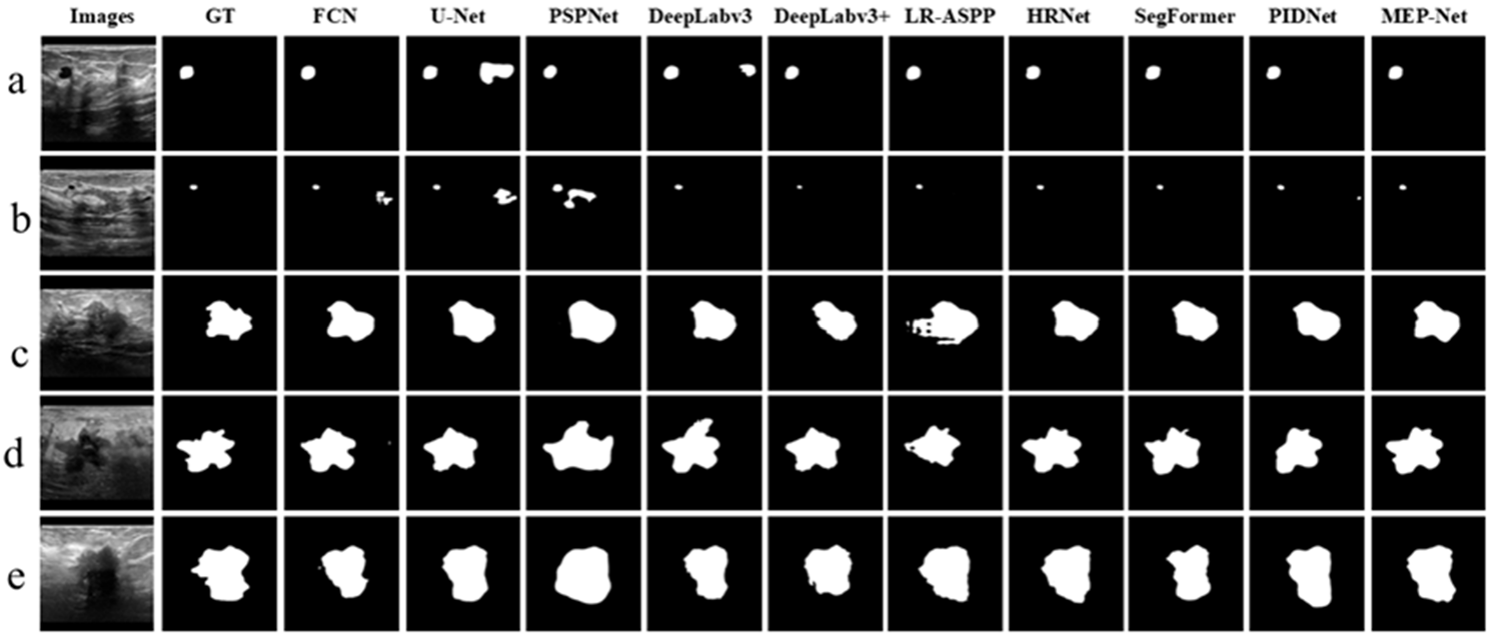
Visual comparison of segmentation results from different models on representative BUSI test images. Each row shows a breast ultrasound image, the corresponding GT mask, and predictions from the compared models

As illustrated in the visual results (Fig. 6), the performance of various segmentation models on breast ultrasound images exhibits notable differences, particularly in their ability to handle blurred boundaries, detect small lesions, and reconstruct complex anatomical structures. Overall, the segmentation outcomes generated by MEP-Net demonstrate closer alignment with the GT, particularly in terms of boundary accuracy and morphological consistency. In Fig. 6(a), the lesion exhibits well-defined edges and moderate size. FCN and DeepLabv3 + tend to over-segment regions beyond the actual lesion, whereas other models produce results more consistent with the GT. MEP-Net effectively suppresses false positives and yields segmentation results that more closely match the GT. In Fig. 6(b), the lesion is small yet clearly delineated. FCN, U-Net, PSPNet, and PIDNet generate several false predictions, while DeepLabv3 + fails to fully capture the lesion region. In this scenario, MEP-Net and several other advanced models achieve more robust segmentation performance, demonstrating improved agreement with the GT in both boundary delineation and morphological preservation. In Fig. 6(c), (d), and (e), the lesions exhibit structural complexity and indistinct boundaries. Although all models are able to localize the lesions, MEP-Net demonstrates higher consistency with the GT in restoring fine details and maintaining boundary continuity.

In general, MEP-Net generates segmentation results on breast ultrasound images that exhibit strong agreement with the GT. Under challenging conditions—such as blurred boundaries and complex lesion morphologies—MEP-Net demonstrates clear advantages over conventional models across multiple evaluation metrics.

#### Computational efficiency analysis

To evaluate the computational efficiency of the proposed method, the model complexity of the baseline PIDNet and the proposed MEP-Net is compared in terms of the number of parameters (Parameters), floating-point operations (FLOPs), and inference time (Inference Time).

**Table 4:** Comparison of computational efficiency among different models

Method	Parameters (M)	FLOPs (G)	Inference Time (ms/img)	FPS
PIDNet	35.47	23.58	334.84	2.99
MEP-Net	35.60	24.50	409.96	2.44

As shown in Table 4, PIDNet has 35.47 M parameters, while MEP-Net has 35.60 M, representing only a 0.37% increase. This indicates that the introduced modules have a minimal impact on model size. In terms of computational complexity, PIDNet requires 23.58 G FLOPs, whereas MEP-Net requires 24.50 G FLOPs, an increase of approximately 3.9%, suggesting only a slight increase in computational cost. For inference efficiency, the forward time per image is measured under the same input size and experimental environment (batch size = 1). The results show that PIDNet achieves an inference time of 334.84 ms/image (approximately 2.99 FPS), while MEP-Net requires 409.96 ms/image (approximately 2.44 FPS). Although MEP-Net introduces a slight increase in inference time, it maintains a comparable runtime speed overall.

In summary, MEP-Net improves segmentation performance with only a marginal increase in parameters (+ 0.37%) and limited additional computational cost (+ 3.9% FLOPs). Despite the increase in inference time, the model still maintains good efficiency, demonstrating a favorable trade-off between accuracy and computational complexity.

#### Ablation study

To validate the effectiveness of the MECS and ECA modules, ablation experiments were conducted by incorporating each module individually into the PIDNet backbone, with the results shown in Table 5. On both datasets, the introduction of either the MECS or ECA module led to an improvement in Recall (CEUS: +1.74%/+2.09%; BUSI: +1.35%/+1.31%). However, the impact on Precision was limited, with MECS even causing a slight decrease in Precision on the BUSI dataset (-0.20%). These results indicate that both attention modules enhance the detection ability of the target region. This aligns with the observed trade-off between Precision and Recall in the overall experiments.

Specifically, the MECS module improved Recall (CEUS: +1.74%, BUSI: +1.35%) and Dice (CEUS: +1.14%, BUSI: +0.65%) on both datasets, demonstrating its effectiveness in enhancing the model’s coverage of lesion regions. In contrast, the ECA module showed an advantage in controlling false positives, with a greater improvement in MIoU on the CEUS dataset (+ 0.85%) compared to MECS (+ 0.13%). It also boosted Precision on both datasets. When MECS and ECA were used together, MEP-Net achieved the best results in Recall and Dice, with no significant drop in Precision. This suggests that the two modules are complementary: MECS primarily enhances region coverage, while ECA suppresses false positives to some extent.

It is noteworthy that, even when used together, MEP-Net’s Precision improvement on the BUSI dataset was only + 0.08%, much lower than the improvement in Recall (+ 2.41%). This indicates that while the model reduces false negatives, the improvement in false positives is relatively limited. This phenomenon reflects the higher difficulty in discriminating some samples in the BUSI dataset, which leaves room for further optimization. Overall, both MECS and ECA bring performance gains individually, and their combined use yields the best results. However, there is still room for improvement in false positive control.

**Table 5:** Performance of PIDNet with different attention modules on the CEUS and BUSI datasets (mean ± SD, improvement over baseline)

Dataset	Model	+MECS	+ECA	Precision (%)	Recall (%)	MIoU (%)	Dice (%)
**CEUS**	**PIDNet**	×	×	81.42 ± 0.96 (+ 0.00)	65.16 ± 1.28 (+ 0.00)	77.71 ± 0.31 (+ 0.00)	72.38 ± 0.49 (+ 0.00)
		✓	×	81.64 ± 2.03 (+ 0.22)	66.90 ± 1.14 (+ 1.74)	77.84 ± 0.29 (+ 0.13)	73.52 ± 0.43 (+ 1.14)
		×	✓	81.61 ± 1.40 (+ 0.21)	67.25 ± 1.72 (+ 2.09)	78.56 ± 0.86 (+ 0.85)	73.71 ± 0.49 (+ 1.33)
		✓	✓	**82.43 ± 0.74** **(+ 1.03)**	**67.67 ± 0.39** **(+ 2.51)**	**78.96 ± 0.25** **(+ 1.25)**	**74.33 ± 0.38** **(+ 1.95)**
**BUSI**	**PIDNet**	×	×	85.87 ± 1.70 (+ 0.00)	76.15 ± 1.60 (+ 0.00)	82.13 ± 1.21 (+ 0.00)	80.71 ± 1.55 (+ 0.00)
		✓	×	85.67 ± 0.97 (-0.20)	77.50 ± 2.28 (+ 1.35)	82.62 ± 0.63 (+ 0.49)	81.36 ± 0.83 (+ 0.65)
		×	✓	**86.32 ± 0.13** **(+ 0.45)**	77.46 ± 0.29 (+ 1.31)	82.86 ± 0.17 (+ 0.73)	81.65 ± 0.22 (+ 0.94)
		✓	✓	85.95 ± 1.45 (+ 0.08)	**78.56 ± 0.57** **(+ 2.41)**	**83.19 ± 0.41** **(+ 1.06)**	**82.08 ± 0.49** **(+ 1.37)**

## Discussion

The proposed MEP-Net model achieves improved performance compared to the baseline PIDNet for CEUS liver tumor image segmentation. It yields modest but noticeable gains in both Dice coefficient and Recall. The experimental results suggest that this improvement may stem from the introduced median-enhanced channel-space attention module and the efficient channel attention module. The MECS module constructs channel descriptions from different statistical perspectives by combining global average pooling, max pooling, and median pooling, which captures the intermediate trend information of the channel response distribution. Its multi-scale spatial modeling structure enhances the model’s ability to represent lesions of various shapes. The ECA module, with its lightweight design, facilitates inter-channel information interaction and improves the discriminative ability of fused features while maintaining low computational cost.

Ablation study results further confirm that both types of attention mechanisms contribute to performance gains to varying degrees across the two datasets, supporting their effectiveness in ultrasound image segmentation tasks. In addition, the model exhibits relatively stable performance on the BUSI dataset, suggesting some level of cross-dataset generalization. Considering the differences between BUSI and CEUS data in imaging mechanisms, lesion characteristics, and image quality, these results mainly reflect the model’s performance under the evaluated data conditions.

It should be further emphasized that this study focuses primarily on image segmentation performance, and the evaluation metrics are limited to pixel-level segmentation accuracy. The study does not involve assessment of diagnostic accuracy, treatment planning, or clinical outcomes. Therefore, the results should not be directly interpreted as having an impact on clinical diagnosis or treatment workflows. Instead, this work is intended to provide methodological insights for future related research.

Despite the initial improvements in segmentation accuracy, the MEP-Net model still exhibits several limitations. (1) The CEUS dataset used in this study is mainly collected from a single center, resulting in a relatively concentrated data distribution. Validation on multi-center datasets is still needed. (2) The model shows limited sensitivity to small or low-contrast lesions in some images [36]. This may be related to insufficient capture of local information during feature fusion. (3) The evaluation metrics used in this study are based on pixel-level accuracy. However, clinical diagnosis often relies on temporal information, such as dynamic enhancement patterns and morphological evolution [37, 38]. (4) The current experiments were primarily conducted under standard image quality conditions, and the model’s performance under degraded conditions, such as speckle noise, image blur, or reduced contrast, has not been systematically evaluated. Therefore, the median-based statistical design of the MECS module should currently be viewed as a feature enhancement strategy aimed at the complex grayscale distribution of CEUS images. Its robustness under degraded conditions still needs to be further validated in future studies. (5) The overall performance improvement over the baseline model is relatively modest, indicating that further optimization is still needed.

In addition, this study employs a key-frame extraction strategy based on grayscale variation, which effectively removes static redundant frames and focuses on the key temporal phases of contrast agent dynamics. However, compared to uniform and random sampling, this method tends to prioritize frames with significant grayscale fluctuations, which may introduce selection bias and temporal sampling imbalance. This could potentially impact the model’s generalization ability. Due to the limitations of this study, a comparative experiment of multiple sampling strategies has not been conducted, making it difficult to quantitatively assess the impact of this bias on model performance. Future research will compare different frame sampling strategies (such as uniform and random sampling) to validate the stability and generalization capability of this approach.

Overall, MEP-Net demonstrates stable segmentation performance under the datasets and experimental settings used in this study. By introducing attention mechanisms tailored to ultrasound image characteristics, the model achieves improvements in lesion-related feature modeling and multi-branch feature fusion, providing a useful reference for automatic HCC segmentation in CEUS images. Future work will further evaluate the model under broader conditions, including multi-center datasets, different frame sampling strategies, and video-based sequence modeling.

## Conclusion

This study proposes an improved method for hepatocellular carcinoma (HCC) lesion segmentation, termed MEP-Net. The method builds upon PIDNet and incorporates a median-enhanced channel–spatial attention (MECS) module and an efficient channel attention (ECA) module. These designs aim to improve feature representation and fusion, thereby enhancing lesion segmentation performance in ultrasound images. The proposed model is validated on two datasets, including a self-collected CEUS dataset and the publicly available BUSI dataset. Experimental results show that MEP-Net outperforms the baseline PIDNet and several widely used semantic segmentation models across multiple evaluation metrics. These findings indicate that the proposed method can effectively improve segmentation quality of lesion regions under the evaluated data conditions.

Overall, MEP-Net demonstrates strong segmentation performance under the experimental settings of this study and provides a feasible approach for automatic HCC segmentation in CEUS images. Future work will further validate and refine the model using multi-center datasets and more comprehensive evaluation settings.

## Data Availability

The ultrasonic contrast-enhanced imaging dataset used in this study was obtained from the Department of Ultrasound, Eastern Hepatobiliary Surgery Hospital of the Naval Medical University of the People’s Liberation Army of China. Due to patient privacy and ethical constraints, and the need for future research purposes, the original data cannot be made public.
